# Obesity and Fatty Acids Promote Mitochondrial Translocation of STAT3 Through ROS-Dependent Mechanisms

**DOI:** 10.3389/fragi.2022.924003

**Published:** 2022-07-19

**Authors:** Rachel Conway, Jack Donato Rockhold, Sara SantaCruz-Calvo, Emelia Zukowski, Gabriella H. Pugh, Hatice Hasturk, Philip A. Kern, Barbara S. Nikolajczyk, Leena P. Bharath

**Affiliations:** ^1^ Department of Nutrition and Public Health, Merrimack College, North Andover, MA, United States; ^2^ Department of Pharmacology and Nutritional Sciences, University of Kentucky, Lexington, KY, United States; ^3^ Barnstable Brown Diabetes and Obesity Center, University of Kentucky, Lexington, KY, United States; ^4^ Department of Microbiology, Immunology and Molecular Genetics, University of Kentucky, Lexington, KY, United States; ^5^ Forsyth Institute, Cambridge, MA, United States; ^6^ Department of Medicine, University of Kentucky, Lexington, KY, United States

**Keywords:** mitochondrial STAT3, inflammation, T cells, cytokines, ROS, peroxide, obesity

## Abstract

Obesity promotes the onset and progression of metabolic and inflammatory diseases such as type 2 diabetes. The chronic low-grade inflammation that occurs during obesity triggers multiple signaling mechanisms that negatively affect organismal health. One such mechanism is the persistent activation and mitochondrial translocation of STAT3, which is implicated in inflammatory pathologies and many types of cancers. STAT3 in the mitochondria (mitoSTAT3) alters electron transport chain activity, thereby influencing nutrient metabolism and immune response. PBMCs and CD4^+^ T cells from obese but normal glucose-tolerant (NGT) middle-aged subjects had higher phosphorylation of STAT3 on residue serine 727 and more mitochondrial accumulation of STAT3 than cells from lean subjects. To evaluate if circulating lipid overabundance in obesity is responsible for age- and sex-matched mitoSTAT3, cells from lean subjects were challenged with physiologically relevant doses of the saturated and monounsaturated fatty acids, palmitate and oleate, respectively. Fatty acid treatment caused robust accumulation of mitoSTAT3 in all cell types, which was independent of palmitate-induced impairments in autophagy. Co-treatment of cells with fatty acid and trehalose prevented STAT3 phosphorylation and mitochondrial accumulation in an autophagy-independent but cellular peroxide–dependent mechanism. Pharmacological blockade of mitoSTAT3 either by a mitochondria-targeted STAT3 inhibitor or ROS scavenging prevented obesity and fatty acid–induced production of proinflammatory cytokines IL-17A and IL-6, thus establishing a mechanistic link between mitoSTAT3 and inflammatory cytokine production.

## Introduction

Signal transducers and activators of transcription family 3 (STAT3) play a significant role in immune responses, cancer development, and cellular metabolism, in part by activating cytokine transcription. Examples include deletion of STAT3 in T cells, which abrogated IL-17 cytokine production to attenuate autoimmunity (Yang et al., 2011). Mechanistic work showed that activation and nuclear translocation of STAT3 induced IL-17A gene expression in T cells responding to co-stimulation through CD28 (Kunkl et al., 2019). Although these studies focused on STAT3’s role as a transcription factor, emerging evidence suggests that the non-canonical functions of STAT3 might be at least as critical for regulating overall cellular outcomes. One of these non-canonical functions of STAT3 seems linked to the import of STAT3 into mitochondria as increasingly appreciated in many cell types over the last decade (Wegrzyn et al., 2009). Mitochondrial STAT3 (MitoSTAT3) regulates electron transport chain (ETC) activity, ATP generation, calcium content, and reactive oxygen species (ROS) production (Wegrzyn et al., 2009), (Meier et al., 2017) and (Rincon and Pereira, 2018). It is unclear how STAT3 translocates into mitochondria, but data indicate that it may be imported through the outer mitochondrial membrane protein Tom20 with the heat shock protein 22 (Hsp22) as an alternative shuttle (Rincon and Pereira, 2018), (Mackenzie et al., 2013), (Qiu et al., 2012). MitoSTAT3 interactions with the GRIM-19 protein in Complex 1 may mediate ETC function and ATP generation (Tammineni et al., 2013).

Most data describing mitoSTAT3 have been generated using malignant cells. Cancer cells lacking phospho-serine (p-Ser) 727 STAT3, a modification known to be critical for mitochondrial translocation, were more sensitive to oxidative stress than intact cells. This effect was independent of tyrosine 705 phosphorylated STAT3, a modification necessary for nuclear translocation and transcriptional function (Cheng et al., 2017) and suggested that p-Ser 727 of STAT3 mitigates harmful ROS responses. p-Ser727 STAT3 also controls mitochondrial respiration and Ras transformation in cancer cell lines (Gough et al., 2009) (Wegrzyn et al., 2009). Numerous data show that mitochondria regulate biosynthesis, intracellular signaling, and bioenergetics and play a central role in the function of all cell types, including immune cells. We posit that mitoSTAT3, through its impact on mitochondrial function, orchestrates the immune response in chronic inflammatory conditions such as obesity and beyond.

In this study, we assessed if mitochondrial translocation of STAT3 occurred in immune cells in response to obesity and, as an *in vivo* mimic of obesity sequalae, high fatty acid challenge. The overarching objective of this study was to identify the potential nutrient-sensitive mechanism(s) that promoted mitochondrial translocation of STAT3, along with functional implications of mitoSTAT3 in immune responses as indicated by cytokine production. New data show that mitochondrial translocation of STAT3 occurred in cells exposed to fatty acid challenge and in cells from adults that are obese but are normal glucose-tolerant (NGT). Translocation was not directly dependent on macroautophagy but instead required cellular peroxides. Pharmacological blockade of mitoSTAT3 either by a mitochondrion-targeted STAT3 inhibitor or ROS scavenger concomitantly prevented obesity-associated and fatty acid–induced production of the proinflammatory cytokines IL-17A and IL-6 to indicate a mechanistic link between mitoSTAT3 and inflammatory cytokine production.

## Materials and Methods

### Human Subject Sample Collection

In accordance with the Declaration of Helsinki, informed consent for all human participants was obtained following the Institutional Review Board–approved protocols at the Forsyth Institute and the University of Kentucky. Peripheral blood was obtained from normoglycemic subjects who were lean (avg: 41.2 years; BMI 23.8 kg/m^2^) or obese/normal glucose-tolerant (NGT; avg: 46.6 years; BMI 39.7 kg/m^2^). A1c or HbA1c is a blood test that measures average blood sugar levels. The A1c test showed that both lean and obese subjects were glucose-tolerant. Additional characteristics are shown in Table 1. Exclusion criteria were smoking, recent use of antibiotics, or anti-inflammatory medications, that is, NSAIDs/steroids, colds/flu within the past 2 weeks, type 2 diabetes or diabetes medications, and any history of cancer, hyperglycemia, and auto-immune diseases.

**TABLE 1:** Description of research subjects.

	Lean	Obese
Total N	12	12
Age, yrs. [ Mean (range)]	41.2 (38.5–57.0)	46.6 (38.3–55.5)
A1c, % [Mean (range)]	5.1 (4.5–5.6)	5.3 (5.0–5.5)
BMI, Kg m^-2^[Mean (range)]^*^	23.8 (19.0–24.4)	39.7 (31.6–48.4)
Females [N (%)]	8 (66%)	8 (66%)
Males [N (%)]	4 (33%)	4 (33%)

* P < 0.05, Student’s T test Lean vs Obese.

### Isolation of PBMCs and T Cells

Fifty milliliter of peripheral blood was collected into acid/citrate/dextrose containing tubes by venous puncture. PBMCs were purified by histopaque and frozen at −80°C under controlled cooling conditions in a Mr. Frosty apparatus (Nalgene). For multi-week storage, cells were moved to −190°C following 1–2 days at −80°C. CD4^+^ T cells were isolated from PBMCs by negative selection using MACS columns (Miltenyi Biotech). PBMCs or isolated CD4^+^ T cells were stimulated *in vitro* for 40 h with T cell–activating αCD3/αCD28 Dynabeads (Thermo Fisher Scientific, 11132D) at 2 μL Dynabeads per 100k cells. In some cultures, cells obtained from lean adults were treated with either 300 µM palmitate (pal) (C16:0) coupled to fatty acid–free bovine serum albumin (BSA) at a ratio of 2 mol palmitate to 1 mol BSA, or 300 µM oleate (Ole). The fatty acid concentrations mimic concentrations achievable in human serum. 1% BSA was used as vehicle (Veh). The following treatments were added to some cultures as indicated: 100 mM trehalose (Tre) or 3 µM Mitocur-1 (Mtcur) for 40 h. The ROS scavenger, tempol (Temp; 100 µM), was added 3 h after Dynabeads to avoid excessive cell death. A minimum of 3 hours without ROS quenchers (i.e., tempol) is required post activation for survival of the cells. Thus, cells remained in Temp for 37 h. The late-stage autophagy blocker bafilomycin (Baf; 1 µM) was added to some cultures treated with Tre for the last 30 min, 1 h, or 4 h of incubation.

All treatments were added to our standard culture media of RPMI with 5 mM glucose (normoglycemic), 10% heat-inactivated FBS, and antibiotics. Supernatants were collected and stored at −80°C and shipped on dry ice to the University of Kentucky for performing the Luminex bio-plex assay to assess the levels of cytokines. Cells were assayed as outlined below.

### Immunofluorescence

*In vitro* activated CD4^+^ T cells obtained from NGT subjects and cells from lean subjects after *in vitro* fatty acid treatments (±Trehalose,± Mtcur,± tempol, ± Baf) were plated on coverslips coated with poly-D-lysine in 6-well or 12-well plates. After 40 h, the cells were briefly centrifuged (1,200 rpm, 10 min), washed two times with ×1 PBS, and incubated in 4% paraformaldehyde for 30 min at RT. The coverslips were washed ×2 with PBS and 0.1% triton X-100 (PBST) and were blocked for at least 30 min in 5% BSA/PBST. Antibodies to TOM20 (Santacruz Biotechnology, Dallas, TX), p-STAT3 Ser 727 (Cell signaling technology, Danvers, MA), and LC3 (Millipore Sigma, Burlington, MA) were added at 1:50 dilution with incubation overnight at 4°C. The coverslips were washed ×2 with PBST and incubated with fluorophore-tagged secondary antibodies (anti-mouse Alexa 488 or anti-rabbit Alexa 680) (Rockland Immunochemicals, Limerick, PA) for 2 h at RT. The coverslips were washed ×2 with PBST and mounted on glass slides using Fluoromount-G (Southern Biotech, Birmingham, AL). Cell imaging under a 63× oil immersion lens was performed in a Zeiss confocal microscope. Seven to ten fields per slide were imaged were imaged on *N* = 3–4 slides, and data were analyzed using ImageJ (Kirber et al., 2007), (Vereb et al., 2000). Pearson’s colocalization coefficient (PCC) is a well-established method that quantifies the degree of overlap between fluorescence in the channels. The PCC is unaffected by changes to the offset and independent of gain. The PCC has a range of +1 (perfect correlation) to −1 (perfect but negative correlation). The PCC is not sensitive to differences in signal intensity between the components of an image caused by different labeling with fluorochromes, photobleaching, or different settings of amplifiers (Adler and Parmryd, 2010).

### Immunoblotting

Immunoblotting quantified protein expression was published (Bharath et al., 2020). Briefly, 30 μl of ×1 cell lysis buffer (Cell signaling technology, Danvers, MA) was added to 1 × 10^6^ cells and incubated on ice for 20 min. Cells were centrifuged at 13,000 rpm for 20 min, and the supernatant was collected. A bicinchoninic assay (Thermo Fisher Scientific, 23225) was used to assess the protein concentration. Fifteen μg protein was loaded onto polyacrylamide gels, and electrophoresis was performed at 100 V for 1 h. The transfer of protein to the polyvinylidene difluoride (PVDF) membrane was performed at 35 V for 5 h. The membrane was blocked for 30 min at room temperature (RT) in the blocking buffer containing 2% bovine serum albumin in TBST followed by overnight incubation at 4^○^C in the respective primary antibodies. The membrane was washed ×3 with 1X TBST and incubated with secondary antibodies for 2 h at RT and then imaged. Table 2 lists the antibodies used in this study. All antibodies were used at a dilution of 1:500 except β-actin, which was used at 1:10,000. We quantified protein expression on Western blots using Image studio lite (Licor, Lincoln, NE).

**TABLE 2:** Reagent or resource.

Antibodies	Source	Identifier
Anti-LC3 used at 1:50 for confocal microscopy	Millipore Sigma	Cat# L7543; RRID:AB_796155
Anti-TOM20 used at 1:50 for confocal microscopy	Santa Cruz Biotechnology	Cat# sc-17764; RRID:AB_628381
Anti-p-STAT3 Ser 727 used at 1:500 for WB, 1:50 for confocal microscopy	Cell signaling technology	Cat# 9134; RRID:AB_331589
Anti-STAT3 used at 1:500	Cell signaling technology	Cat# 4904; RRID:AB_331269
β-actin used at 1:500 for WB	Cell signaling technology	Cat# 3700; RRID: AB_2242334
Anti-mouse Alexa 488 used at 1:500	Rockland antibodies	Cat# 610–741–124; RRID:AB_1057558
Anti-rabbit Alexa 647 used at 1:500	Thermo fisher scientific	Cat# A-21244, RRID:AB_2535812
Anti-mouse IgG, HRP-linked used at 1:5000	Cell signaling technology	Cat# 7076, RRID:AB_330924
Anti-rabbit IgG, HRP-linked used at 1:5000	Cell signaling technology	Cat# 7074, RRID:AB_2099233
Biological Samples		
Lean normal glucose tolerant adults, PBMCs, and CD4^+^ T cells Table 1	This paper	N/A
Obese normal glucose tolerant adults PBMCs and CD4^+^ T cells Table 1	This paper	N/A
Chemicals, peptides, and recombinant proteins		
Bafilomycin A1	Cell signaling technology	Cat# C54645S
Bovine Serum Albumin	Sigma Aldrich	Cat# A8806
2′,7’ –dichlorofluorescin diacetate (DCFDA)	Thermo fisher Scientific	Cat# C6827
Dihydroethidium	Thermo fisher Scientific	Cat#D23107
Mitocur-1	Enamine	Cat# EN300-188280
MitoSOX Red	Thermo fisher Scientific	Cat#M36008
Oleate	Sigma Aldrich	Cat # 01383
Palmitic acid	Sigma Aldrich	Cat# P0500
Tempol	Sigma Aldrich	Cat# SML0737
Trehalose	Sigma Aldrich	Cat# T0167
tert-butyl hydrogen peroxide	Sigma Aldrich	Cat# 416665
Critical Commercial Assays		
Milliplex human Th17 25-plex kit	Millipore Sigma	Cat# HT17MG-14K-PX25
Software and algorithms		
GraphPad Prism version 7 for Windows	GraphPad software	www.graphpad.com
Other		
Dynabeads Human T-Activator CD3⁄CD28 for T Cell Activation	GIBCO life technologies	Cat# 11132D
Human CD4^+^ Isolation kit	Miltenyi	Cat# 130–096–533
CD4^+^ Isolation Columns	Miltenyi	Cat# 130–042–401
RPMI, no glucose	Thermo fisher technologies	Cat# 11879020

### ROS Quantification

ROS was quantified using 10 µM chromomethyl 2′,7′ –dichlorofluorescin diacetate (CM-H_2_DCFDA), fluorescence (Sigma-Aldrich, D6883), 5 µm MitoSox red (Thermo Fisher Scientific, M36008), and 10 µM dihydroethidium fluorescence (DHE) (Thermo Fisher Scientific, D11347) and normalized to cell number. 50 µM tert-butyl hydrogen peroxide (TBHP) (Sigma Aldrich, 416665) was used as a positive control for DCFDA assay. ROS was quantified in the activated PBMCs from NGT subjects and lean subjects after fatty acid treatments (± Tre and ± Temp).

### Cell Viability

Cell viability was assessed via the Alamar blue assay and trypan blue exclusion according to the manufacturer’s directions after different treatments.

### Cytokine Assay

Cytokine production was assessed in supernatants by the Luminex multiplex assay (Milliplex human Th17 25-plex kit, Millipore) and by an enzyme-linked immunosorbent assay (Quantikine ELISA, R&D systems, D1700 and D6050) as we published (Bharath et al., 2020). Outcomes from wells with <35 beads read for each analyte were excluded from the analysis. The plates were washed between incubations using a BioTek 406 Touch plate washer (BioTek) and read using the Luminex FlexMap 3D system (Luminex).

### Statistical Analysis

Data are presented as the mean ± standard error of the mean (SEM). The nonparametric Kruskal–Wallis and Dunn’s post hoc tests or one-way ANOVA (for cytokines) followed by Bonferroni post hoc analysis were performed using Graph-Pad Prism 7.03 (GraphPad Software). The Mann–Whitney test compared means of two values. Significance was accepted when *p* < 0.05.

## Results

### Dietary Fatty Acids Promote Ser-727 Phosphorylation and Mitochondrial Translocation of STAT3

**FIGURE 1:**
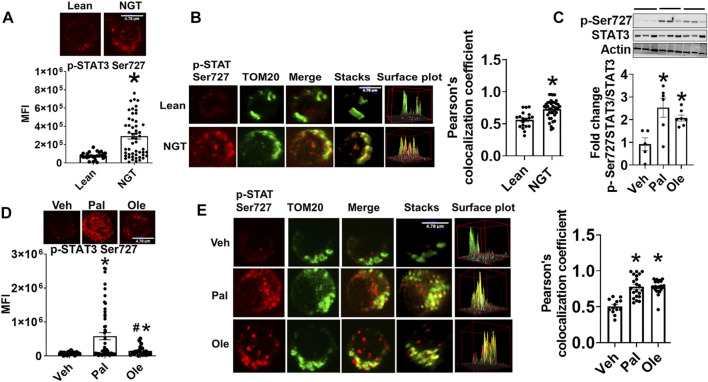
Obesity and excessive fatty acids induce mitochondrial accumulation of p-Ser 727- STAT3. **(A)** p-Ser727 STAT3 quantification in CD4^+^ T cells from lean or obese/normal glucose-tolerant (NGT) subjects. **(B)** Localization of p-Ser727 STAT3 (red) and the Mitochondrial OM protein TOM 20 (green) in CD4^+^ T cells from lean or NGT subjects. Left panel shows representative images and right panel shows quantification of multiple cells. **(C)** p-Ser727 STAT3 expression in PBMCs after treatment with fatty acids assessed by immunoblotting **(D)** p-Ser727 STAT3 expression and **(E)** mitochondrial localization of p-Ser727 STAT3 (red) and the mitochondrial OM protein TOM 20 (green) in CD4^+^ T cells from lean subjects after fatty acid treatment. Left panel shows representative images and right panel shows quantification of multiple cells. *N* = 7–8, **(A,B,D,E)** and *N* = 5, **(C)** Each N represents cells obtained from one subject. At least 7–10 fields per slide were imaged at 63× magnification with oil immersion, on a Zeiss LSM 800 confocal microscope. In fields where numerous cells/fields were observed, the averages are plotted. The brightness of the images was enhanced to improve clarity. The “control” representative images for lean and NGT (Panel 1B) are repeated in panels (Figure 2B, Figure 3B, and Figure 7E). Similarly, the representative images for Veh, Pal, and Ole (1E) are repeated in panels (Figure 4B, Figure 4E, and Figure 7F) *p < 0.05 vs lean or veh, #*p* < 0.05 vs palmitate treatment.

P-Serine (p-Ser) 727 STAT3, a modification required for the translocation of STAT3 into mitochondria, was assessed in CD4^+^ T cells and PBMCs from NGT middle-aged adults (Table 1). We detected significantly more p-Ser 727 STAT3 (Figure 1A) and higher mitochondrial accumulation of STAT3 (Figure 1B) in CD4^+^ cells from NGT adults. To model the possibility that obesity-induced excess in circulating fatty acids promotes mitoSTAT3 accumulation, cells from lean subjects were treated with a physiologically relevant dose of palmitate or oleate prior to analysis. Palmitate induced a robust increase in p-Ser 727 STAT3 (Figure 1C, in PBMCs) in the cytoplasm and the nucleus of CD4^+^ T cells, as evidenced by fluorescence observed in the periphery of the cells and p-Ser 727 STAT3 fluorescence merging with the nuclear stain (data not shown) in a confocal analysis (Figure 1D). Nuclear stain in general is not shown in the images to delineate the STAT3 fluorescence. Confocal microscopy indicated more colocalization of p-Ser-727 STAT3 with mitochondrial translocase of the outer protein membrane, TOM20, following palmitate or oleate treatment of CD4^+^ T cells from lean subjects (Figure 1E). Cell viability was not affected by treatment with fatty acids (Supplementary Figure S3). Collectively, these data support the interpretation that mitochondrial translocation of STAT3 occurs in response to a challenge by saturated and monounsaturated fatty acids *in vitro*, with likely parallels *in vivo*.

### Preventing Mitochondrial Translocation of p-Ser727 STAT3 Abrogates Proinflammatory Cytokine Production

**FIGURE 2:**
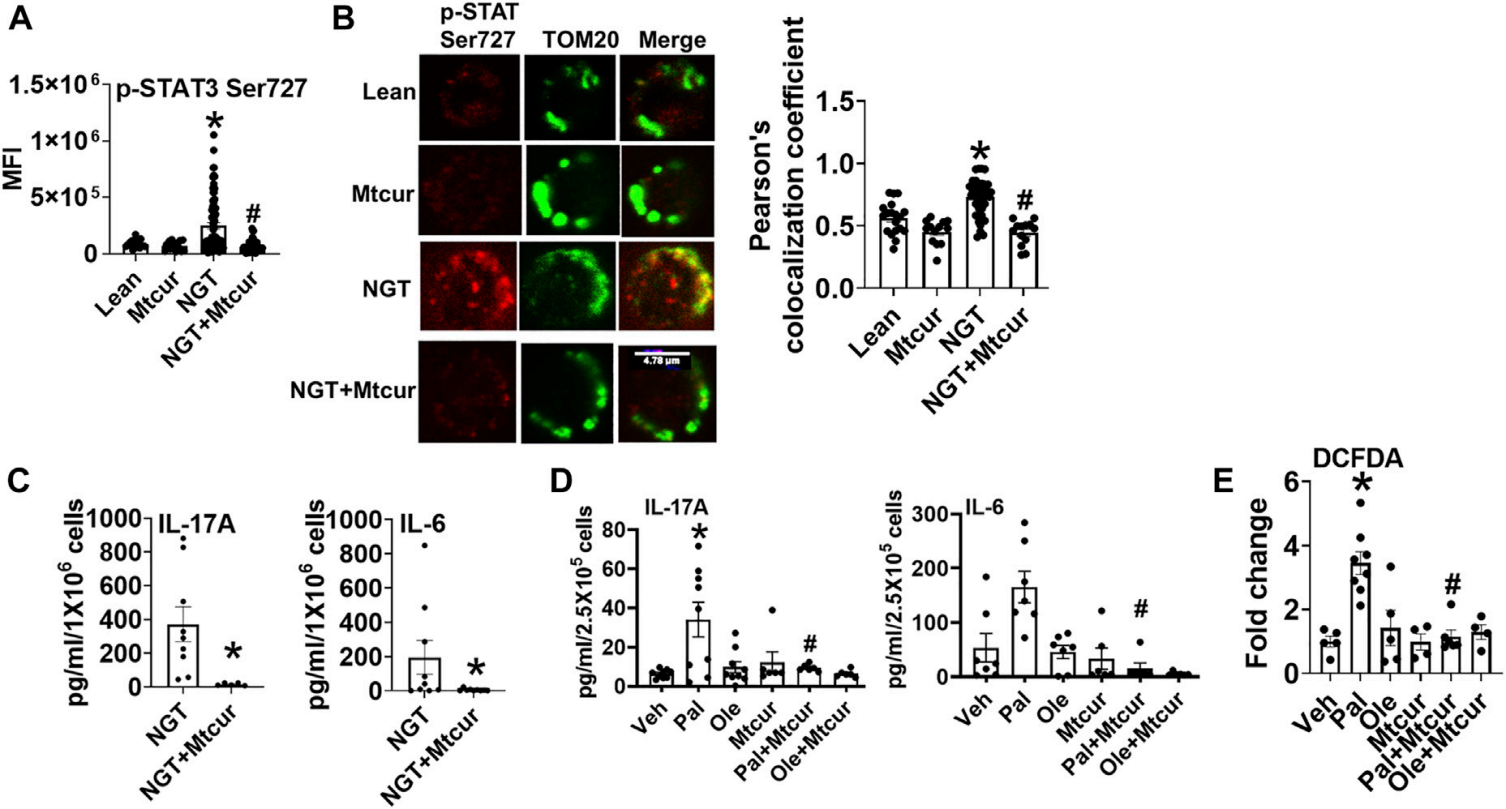
Blocking p-Ser 727- STAT3 translocation into mitochondria prevents proinflammatory cytokine production. **(A)** Expression and **(B)** mitochondrial localization (left panel**,** representative images, and right panel quantification) of p-Ser 727 STAT3 in cells from lean and NGT subjects **(C)** Cytokine production in cells from NGT subjects; **(D)** cytokine production in cells from lean subjects after Mtcur as measured by bio-plex assay. **(E)** Cellular peroxide production. N = 4–8, **(A,B)** and N = 7–9, **(C,D)**. N = 5–8, E. Each N represents cells obtained from one subject. At least 7–10 fields per slide were imaged at 63× magnification with oil immersion, on a Zeiss LSM 800 confocal microscope. In fields where numerous cells/fields were observed, the averages are plotted. The brightness of the images was enhanced to improve clarity. *p < 0.05 vs lean or veh, #*p* < 0.05 vs palmitate treatment. *p < 0.05 vs lean or veh, #*p* < 0.05 vs NGT or palmitate treatment.

To assess the functional consequence of the blocking mitochondrial translocation of STAT3, cells were incubated for 40 h with the mitoSTAT3 inhibitor Mtcur. Lower phosphorylation (Figure 2A), mitochondrial colocalization (Figure 2B), and lower levels of proinflammatory cytokine production (Figure 2C) were observed in mitocur-treated cells from NGT and in lean cells co-treated with Mtcur and palmitate (Figure 2D). Mtcur did not alter cytokine production in cells treated with oleate (Figure 2C). The bio-plex assay indicated Mtcur did not uniformly lower cytokine production (Supplementary Figure S1). These data support the conclusion that mitochondrially located STAT3 promotes inflammation in NGT adults and in palmitate-treated cells from lean subjects.

### Trehalose Prevents Mitochondrial Localization of p-ser727 STAT3 and Lowers Inflammation in NGT Cells

**FIGURE 3:**
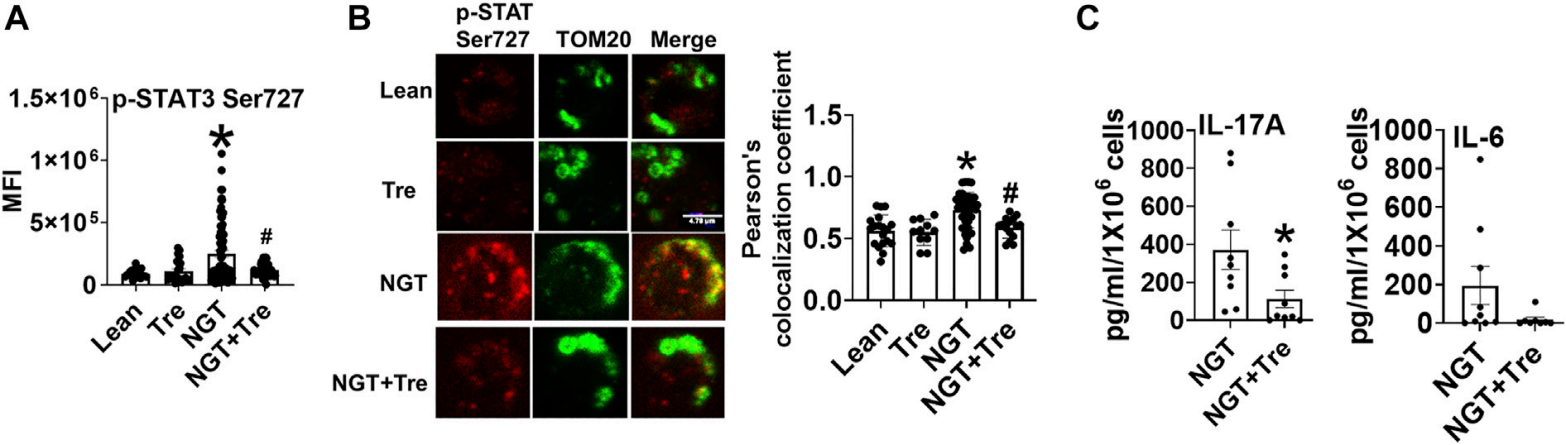
Nutraceutical trehalose blocks p-Ser 727- STAT3 –mitochondrial translocation and lowers inflammation in NGT. **(A)** p-Ser-727 STAT3 expression; **(B)** mitochondrial localization (left panel**,** representative images, and right panel quantification); and **(C)** cytokine production as measured by bio-plex assay by cells from NGT subjects after trehalose treatment. N = 4–7, **(A,B)** and N = 7–9, **(C)**. Each N represents cells obtained from one subject. At least 7–10 fields per slide were imaged at 63× magnification with oil immersion, on a Zeiss LSM 800 confocal microscope. In fields where numerous cells/fields were observed, the averages are plotted. The brightness of the images was enhanced to improve clarity. *p < 0.05 vs lean or NGT, #*p* < 0.05 vs NGT.

**FIGURE 4:**
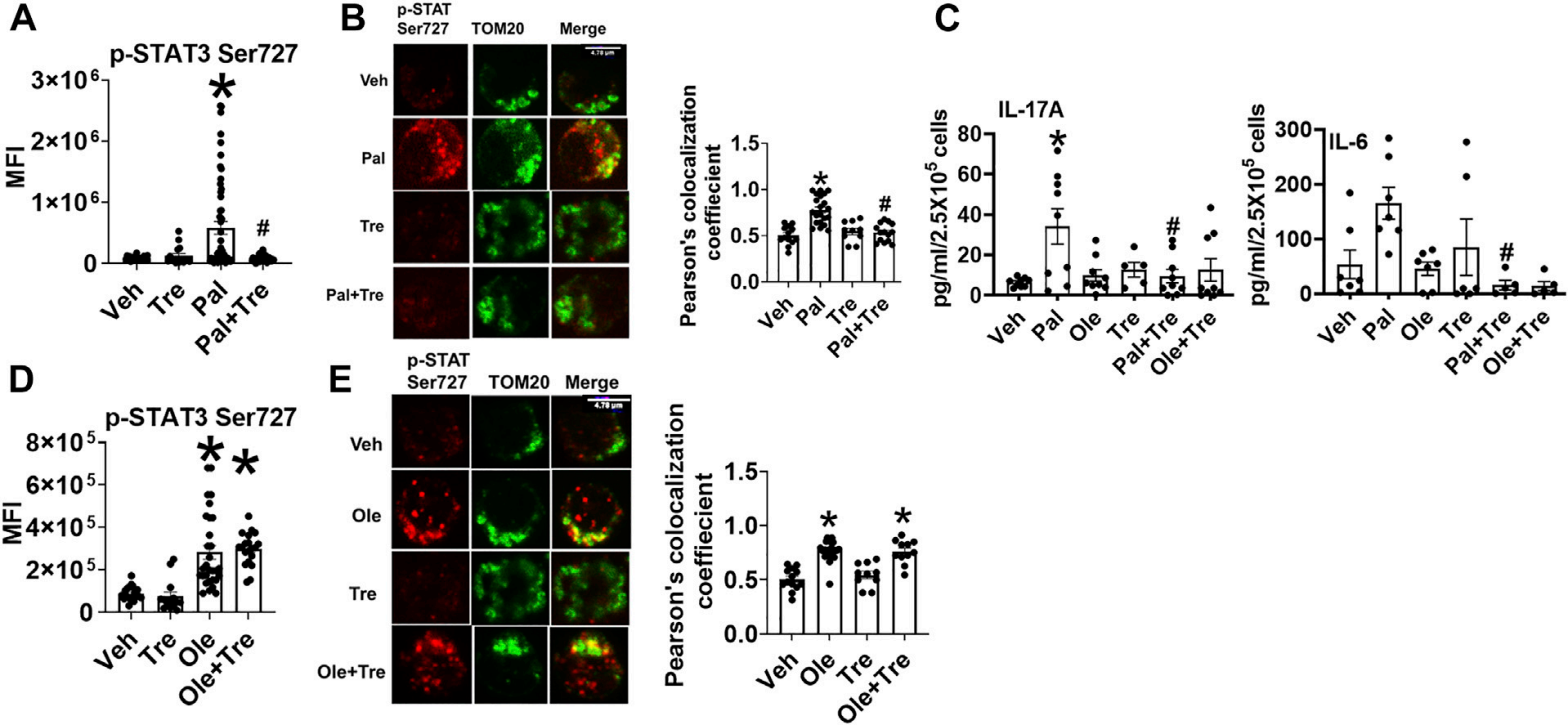
Nutraceutical trehalose blocks p-Ser 727- STAT3 mitochondrial translocation and lowers inflammation in palmitate but not oleate-treated cells. **(A)** p-Ser-727 STAT3 expression; **(B)** mitochondrial localization (left panel representative images and right panel quantification); and **(C)** cytokine production in cells after palmitate and trehalose treatment. **(D)** p-Ser727STAT3 expression and **(E)** mitochondrial localization (left panel, representative images, and right panel quantification) after oleate and trehalose treatment. N = 5–8 for panels **(A,B,D,E)** and *N* = 7–9 for panel **(C)**. Each N represents cells obtained from one subject. At least 7–10 fields per slide were imaged at 63× magnification with oil immersion, on a Zeiss LSM 800 confocal microscope. In fields where numerous cells/fields were observed, the averages are plotted. The brightness of the images was enhanced to improve clarity. *p < 0.05 vs. lean or NGT, #*p* < 0.05 vs. NGT.

To identify the mechanism(s) that promote mitochondrial localization of STAT3, we assessed the processes that are known to promote similar STAT3 translocation in other cell types, including the cellular recycling process of autophagy. We initially tested the effect of trehalose, a nutraceutical known to enhance autophagy on some cell types, on STAT3 translocation and cytokine production. Trehalose treatment of cells from NGT adults prevented obesity-induced phosphorylation and mitochondrial translocation of STAT3, while lowering inflammatory cytokine production (Figures 3A–C). Trehalose also prevented palmitate-induced Ser 727 STAT3 phosphorylation (Figure 4A), STAT3 mitochondrial translocation (Figure 4B), and inflammation (Figure 4C) but was unable to prevent oleate-induced STAT3 phosphorylation and mitochondrial translocation (Figures 4D,E). Supplementary Figure 2A shows the production of other cytokines in response to palmitate and oleate ± trehalose. We conclude that trehalose lowers STAT3-mitochondrial translocation and Th17-associated cytokine production in T cells exposed to saturated but perhaps not monounsaturated lipid excess.

### Trehalose Does not Impact Palmitate-Induced Activation and Translocation of STAT3 by Promoting Autophagy

**FIGURE 5:**
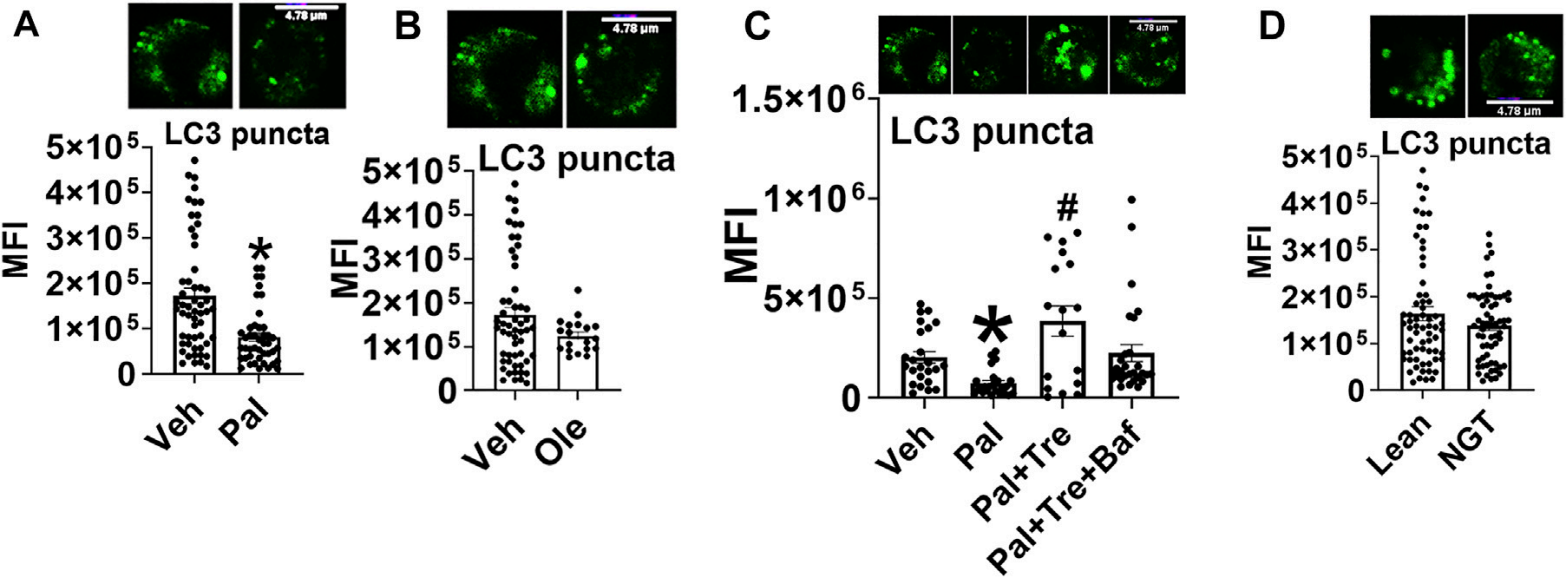
Trehalose does not impact palmitate-induced p-Ser 727- STAT3 and mitochondrial translocation by promoting autophagy. **(A)** LC3 puncta after palmitate, **(B)** oleate, **(C)** autophagy flux analysis, and **(D)** puncta in cells from NGT subjects N = 3–4. Each N represents cells obtained from one subject. At least 7–10 fields per slide were imaged at 63× magnification with oil immersion, on a Zeiss LSM 800 confocal microscope. In fields where numerous cells/fields were observed, the averages are plotted. The brightness of the images was enhanced to improve clarity. *p < 0.05 vs. Veh, #*p* < 0.05 vs. Pal.

To identify how the pro-autophagy actions of trehalose account for functions shown in Figure 4, we assessed autophagy in cells activated ± trehalose by immunofluorescence and staining for microtubule associate protein-1 light chain 3B (LC3B) punctuate structures (puncta). Palmitate reduced LC3 puncta formation (Figure 5A), but oleate did not (Figure 5B), indicating saturated but not monounsaturated fatty acids compromise autophagy. However, the autophagic flux assessment using the late-stage autophagy inhibitor bafilomycin A1 (Baf) showed that trehalose did not enhance palmitate-compromised autophagy but instead stalled the clearance of the autophagosome (Figure 5C). Furthermore, autophagy was not impaired in cells from NGT adults (Figure 5D), showing that the effect of trehalose on STAT3 activation and mitochondrial translocation is not secondary to impaired autophagy. These data also indicate (unsurprisingly) that palmitate treatment does not perfectly mimic obesity-associated changes in CD4^+^ Tcell physiology.

### Trehalose Alters Cellular Peroxide Production, Thus Influencing Redox Balance

**FIGURE 6:**
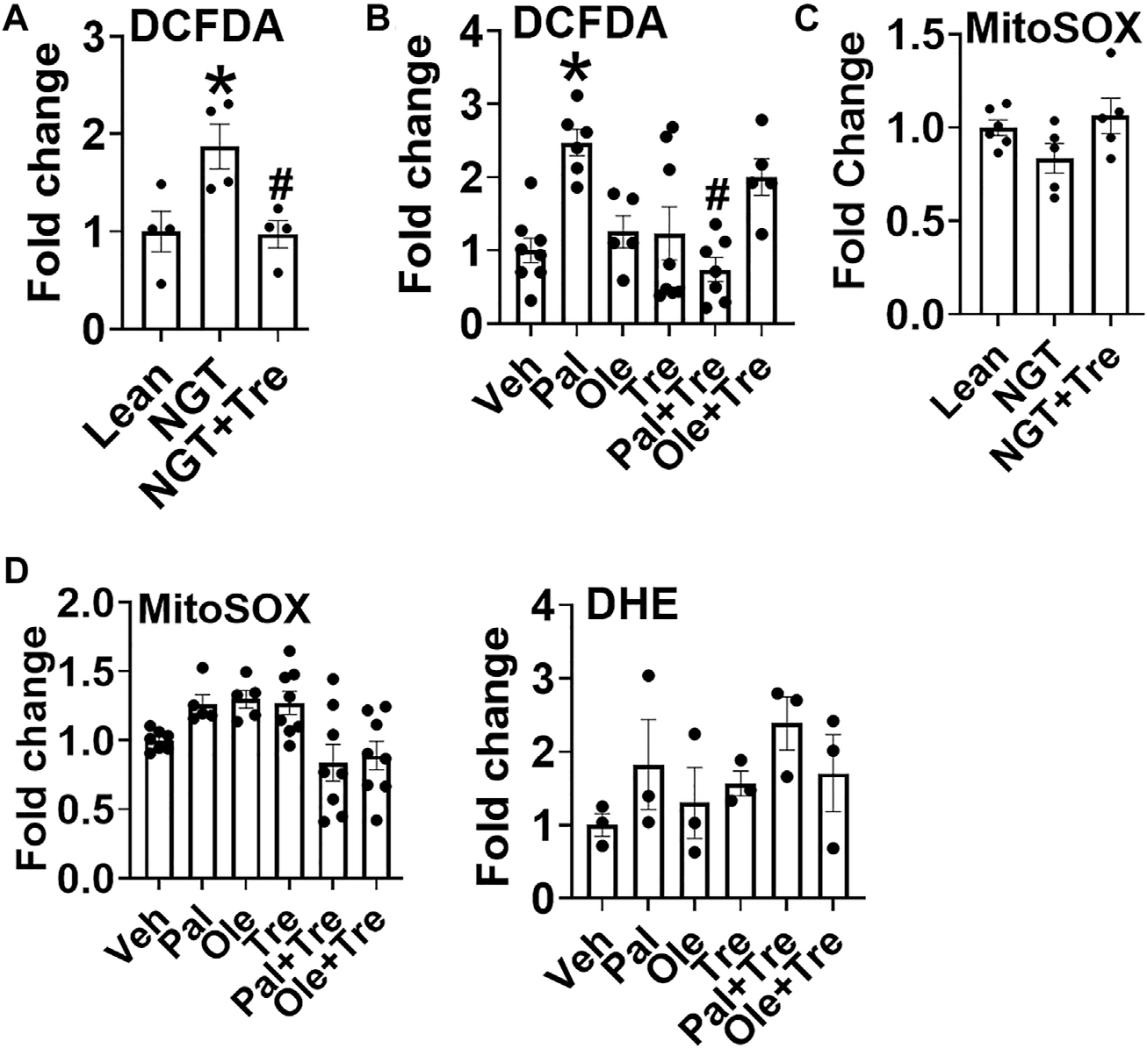
Trehalose lowers cellular peroxide but not superoxide. **(A)** Peroxide production in cells from NGT subjects as measured by DCFDA fluorescence. **(B)** Peroxide production in cells treated as indicated. **(C)** Mitochondrial superoxide production in cells from NGT subjects as measured by MitoSOX. **(D)** Mitochondrial (left panel) and cellular (DHE;right panel) superoxide production in cells treated as indicated**.** N = 3–8, A-D. Each N represents cells obtained from one subject. *p < 0.05 vs. Lean or Veh, #*p* < 0.05 vs. NGT or Pal.

Another well-characterized function of trehalose is its ability to function as an antioxidant, as reported in many studies. Trehalose reduced obesity (Figure 6A) and palmitate-induced (Figure 6B) increase an cellular peroxide levels. Peroxide production did not increase upon oleate treatment; however, a numerical increase in peroxide was observed in cells cotreated with oleate and trehalose (Figure 6B). Neither mitochondrial superoxide nor cellular superoxide was altered in NGT or after treatment with fatty acids, and trehalose did not further affect superoxides in such cultures (Figure 6C and Figure 6D). Together, these data point to the ability of trehalose to lower mitochondrial STAT3 translocation and thus perhaps the production of Th17-associated cytokines by lowering cellular peroxides.

### Lowering Cellular Peroxide Lowers NGT and Pal-Activated pSer727-STAT3-Mitochondrial Translocation and Inflammation

**FIGURE 7:**
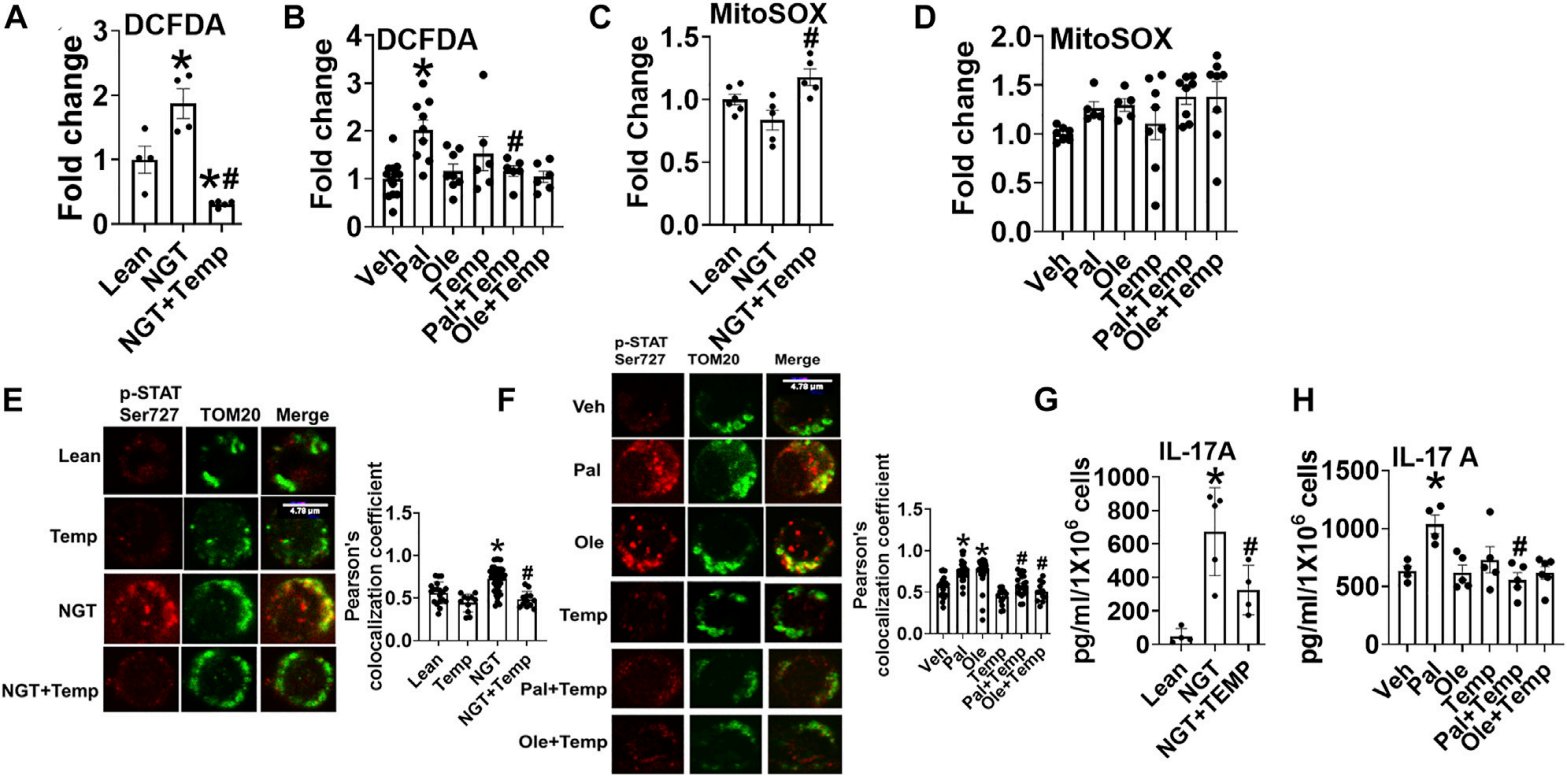
Lowering cellular peroxide with tempol lowers NGT and pal-activated pSer727-STAT3-mitochondrial translocation and inflammation. **(A)** Peroxide production in cells from NGT subjects. **(B)** Peroxide production in cells treated as indicated. **(C)** Superoxide production in cells from NGT subjects. **(D)** Superoxide production in cells treated as indicated. **(E)** p-Ser727 STAT3 expression **(F)**, mitochondrial localization (left panel representative images, and right panel quantification) **(G)** cytokine production in cells from NGT subjects; **(H)** cytokine production by cells treated as indicated, as assessed *via* ELISA assay. N = 5–8, **(A–D)**; *N* = 3–4, **(E,F)**; *N* = 5, **(G-H)**. Each N represents cells obtained from one subject. *p < 0.05 vs. Lean or Veh, #*p* < 0.05 vs. NGT or Pal.

To identify the mechanism that obesity and fatty acids use to promote the mitochondrial translocation of STAT3 and to independently test if trehalose lowers mitoSTAT3 due to the ability to scavenge peroxides, we tested the impact of tempol (Temp), a well-established ROS scavenger, on p-Ser727-STAT3 localization and cytokine production. Temp reduced NGT (Figure 7A) and palmitate-induced peroxide production (Figure 7B). Interestingly, Temp slightly increased mitochondrial superoxide production in NGT (Figure 7C) but had no effect on palmitate- or oleate-treated cells (Figure 7D). Temp prevented mitochondrial translocation of p-Ser 727 STAT3 in cells from NGT adults (Figure 7E) and in both palmitate- or oleate-treated cells (Figure 7F). Temp reduced IL-17 production in cells from NGT (Figure 7G) and palmitate-treated cells from lean subjects (Figure 7H). Collectively, these data show that either scavenging peroxides (but not superoxides) or blocking mitoSTAT3 (Figure 2) prevents IL-17A secretion. We conclude that the mitochondrial translocation of STAT3 favors cellular peroxide formation, which promotes inflammatory cytokine production by T cells primed by high saturated fatty acids.

## Discussion

**FIGURE 8:**
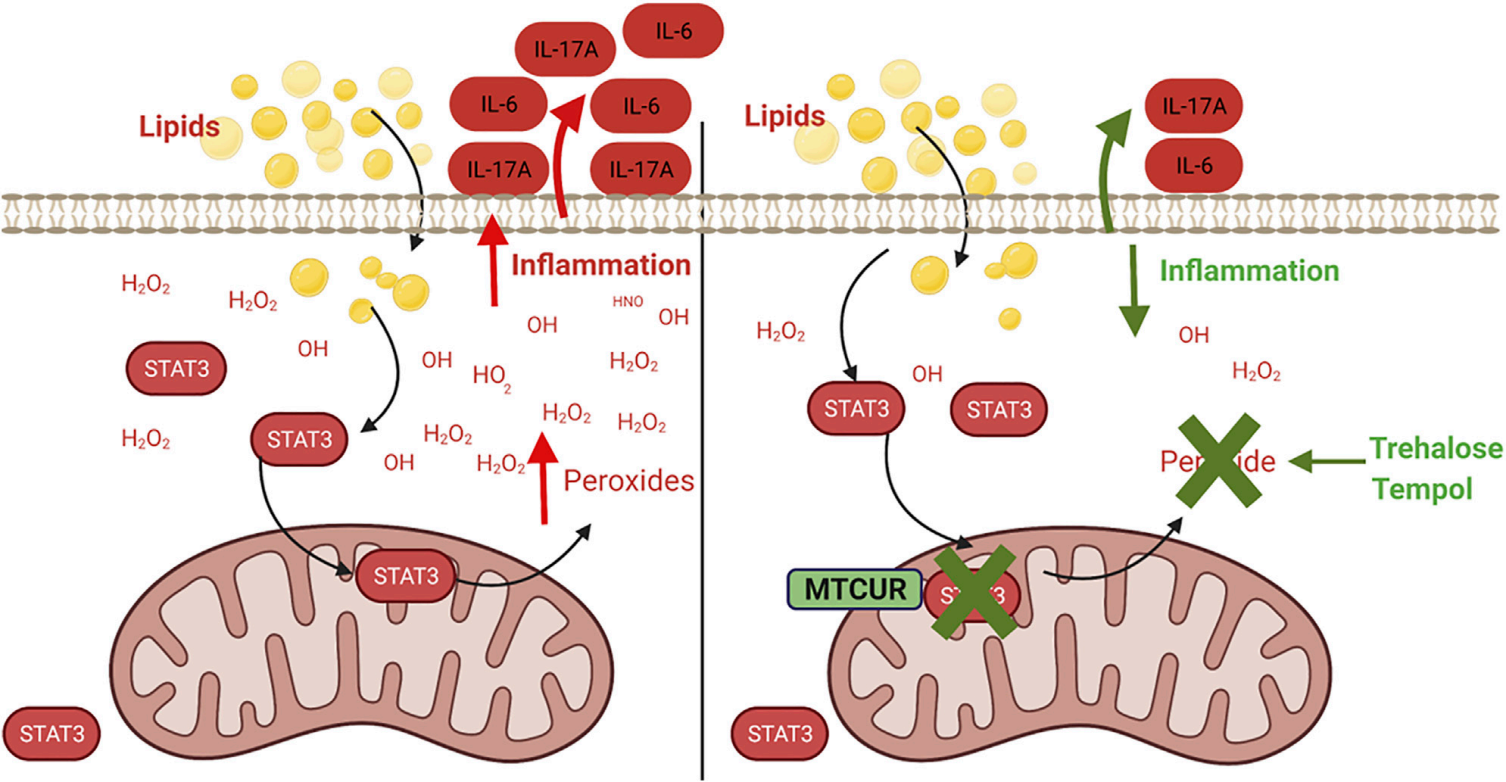
Mitochondrial STAT3 promotes inflammation *via* ROS-dependent mechanisms. Obesity and fatty acid–induced activation and mitochondrial translocation of p-Ser 727-STAT3 (STAT3) promotes peroxide generation within the cell, resulting in the dysregulation of cellular redox balance and promoting inflammatory cytokine production. Preventing accumulation of mitochondrial STAT3 or scavenging of the cellular peroxides alleviates lipid-induced inflammation.

In this study, we provide evidence that obesity and fatty acids promote the mitochondrial translocation of STAT3, which exaggerates cellular peroxide generation and inflammation (Figure 8). We also demonstrate that pharmacological blockade of STAT3 in mitochondria or scavenging of peroxides alter immune cell cytokine secretion, especially preventing the production of IL-17A. The proinflammatory role of IL-17 is recognized in metabolic- and aging-associated diseases (Bechara et al., 2021), (Chen et al., 2020), (Mohammadi Shahrokhi et al., 2018), and our previous work showed that obesity and palmitate partially recapitulated a T2D-associated cytokine profile (McCambridge et al., 2019). In NGT adults, this partial cytokine profile could indicate a physiology along with the continuum of progression from obesity to obesity-associated T2D. In this study, we extend the previous work by identifying a specific cellular mechanism and increased peroxide production that incites this inflammatory milieu and likely sets the stage for developing fulminant T2D and other obesity-associated inflammatory conditions.

During obesity, STAT3, a transcription factor for Th17 cytokines, is chronically activated (Wunderlich et al., 2013). STAT3 upregulation occurs in CD8^+^ T cells during obesity-associated breast tumor progression (Zhang et al., 2020), and T cell STAT3 in turn promotes obesity, insulin resistance, and T2D (Priceman et al., 2013). In addition, elevated leptin, which activates the leptin-Rb-STAT3 signaling axis, may also contribute, although that possibility remains untested. In resting cells, STAT3 is present as an inactive monomer in the cytoplasm. When the cells are activated, STAT3 translocates to the nucleus to initiate transcription by binding to the promoter sequence of the target gene (Liu et al., 2021) (Guendisch et al., 2017). More recently, many studies show the translocation of STAT3 to mitochondria under different conditions (Yang and Rincon, 2016) (Rincon and Pereira, 2018). Recently, the role of mitochondrial STAT3 in IL-6 mediated CD4^+^ T cell motility and stability was also demonstrated, consistent with our findings of upstream regulators of cytokine production (Valença-Pereira et al., 2021). Interestingly, the small portion of STAT3 that migrates to mitochondria exerts profound effects on the cell by modulating the electron transport chain activity and fuel utilization. In mitochondria, STAT3 multitasks by regulating the biological functions of the cell as well as modulating the oncogenic signaling (Ploeger et al., 2020). Phosphorylation at the serine residue (p-Ser 727), enhances its mitochondrial function, and constitutive p-Ser 727, which is known to occur in many human cancers, appears sufficient to drive tumorigenesis in oncogenic models (Zhang et al., 2013), (Gough et al., 2013), (Gough et al., 2009). Studies show that overexpression of FTO, a regulator of the leptin/STAT3 axis mentioned above, decreases the tyrosine phosphorylation of STAT3 but increases the mitochondrial S727 phosphorylation in mouse liver, thus demonstrating that FTO favors mitochondrial translocation at the expense of nuclear translocation. Thus, our data are congruent with the observations in other cell types that mitoSTAT3 occurs in response to obesity [reviewed (Kadye et al., 2020)]. The FTO-induced mitochondrial translocation of STAT3 upregulated neo glucogenic genes and increased mitochondrial density and function in the liver of FTO overexpressing mice, but the possibility is that it also triggers ROS was not queried (Bravard et al., 2014).

ROS, although critical for maintaining cellular homeostasis, is detrimental to cells if produced in excess. Dysregulation of ROS signaling can promote inflammatory and metabolic pathologies, although mechanistic details as our new data provided are often missing (Previte et al., 2017), (McMurray et al., 2016). Our data show the involvement of cellular peroxides in promoting but not mitochondrial or cellular superoxide obesity and fatty acid–induced inflammation. Our results agree with the published data, which showed that the mitochondrion-targeted antioxidant, Mito TEMPO, failed to impact IL-17 A/F cytokine production in cells treated with palmitate (McCambridge et al., 2019). However, the existence of a relationship between mitochondrial redox homeostasis and cytokines cannot be entirely excluded because 1) mitochondrial ROS could happen as a burst at a time point, which could initiate subsequent production of cellular peroxides at other time points, with mitochondrial ROS acting as an initiator, that is , ROS-induced-ROS response (RIRR) (Dmitry, 2014) and 2) mtcur treatment improved mitochondrial nicotinamide nucleotide transhydrogenase (NNT) expression (data not shown), a redox regulator, downregulation of which increased in turn IL-17A/F (McCambridge et al., 2019). From these data, we conclude that a complex ROS signaling mechanism regulates mitochondria. Although obesity and palmitate did not promote mitochondrial ROS in our experimental context, we cannot ignore demonstrations that obesity-induced dysregulation of mitochondrial redox homeostasis may occur outside the time frame of detection, which can result in the accumulation of damage to mitochondria, eventually promoting proinflammatory milieu and the onset of obesity-associated inflammation.

Collectively, our data show that obesity and fatty acid promote activation and mitochondrial translocation of p-Ser 727 STAT3, resulting in exaggerated peroxide and increased cytokine production. We have thus identified a mechanistic link between mitoSTAT3 and proinflammatory cytokines to underscore the importance of mitoSTAT3 in regulating obesity-associated inflammation for the first time. Our work is limited because 1) we did not evaluate the detailed signaling mechanism through which STAT3 activation and mitochondrial translocation result in cellular peroxide generation and 2) MitoSTAT3 may have a role in T-cell and immune-cell fatty acid oxidation that we did not investigate. Additionally, a recent report indicated that STAT3 is not associated with mitochondria but instead localized with mitochondrion-associated membranes (MAMs), where mitochondria and the endoplasmic reticulum tightly associate (Su et al., 2020). We acknowledge that this is a possibility, and our confocal microscopy–based work may not have sufficiently delineated mitochondria from MAMs and thus needs further evaluation. Detailed investigation of immune cell mitoSTAT3 in response to physiological and pathophysiological challenges, intracellular signaling mechanism(s), and the functional consequences remains as future directions.

## Data Availability

The raw data supporting the conclusion of this article will be made available by the authors, without undue reservation.
