# Supplementary material for: Obesity and Fatty Acids Promote Mitochondrial Translocation of STAT3 Through ROS-Dependent Mechanisms
Source: Front Aging. 2022 Jul 19;3:924003. doi: 10.3389/fragi.2022.924003 (PMC9344057; doi:10.3389/fragi.2022.924003)
Supplement: Supplementary file 1 [file DataSheet1.docx]

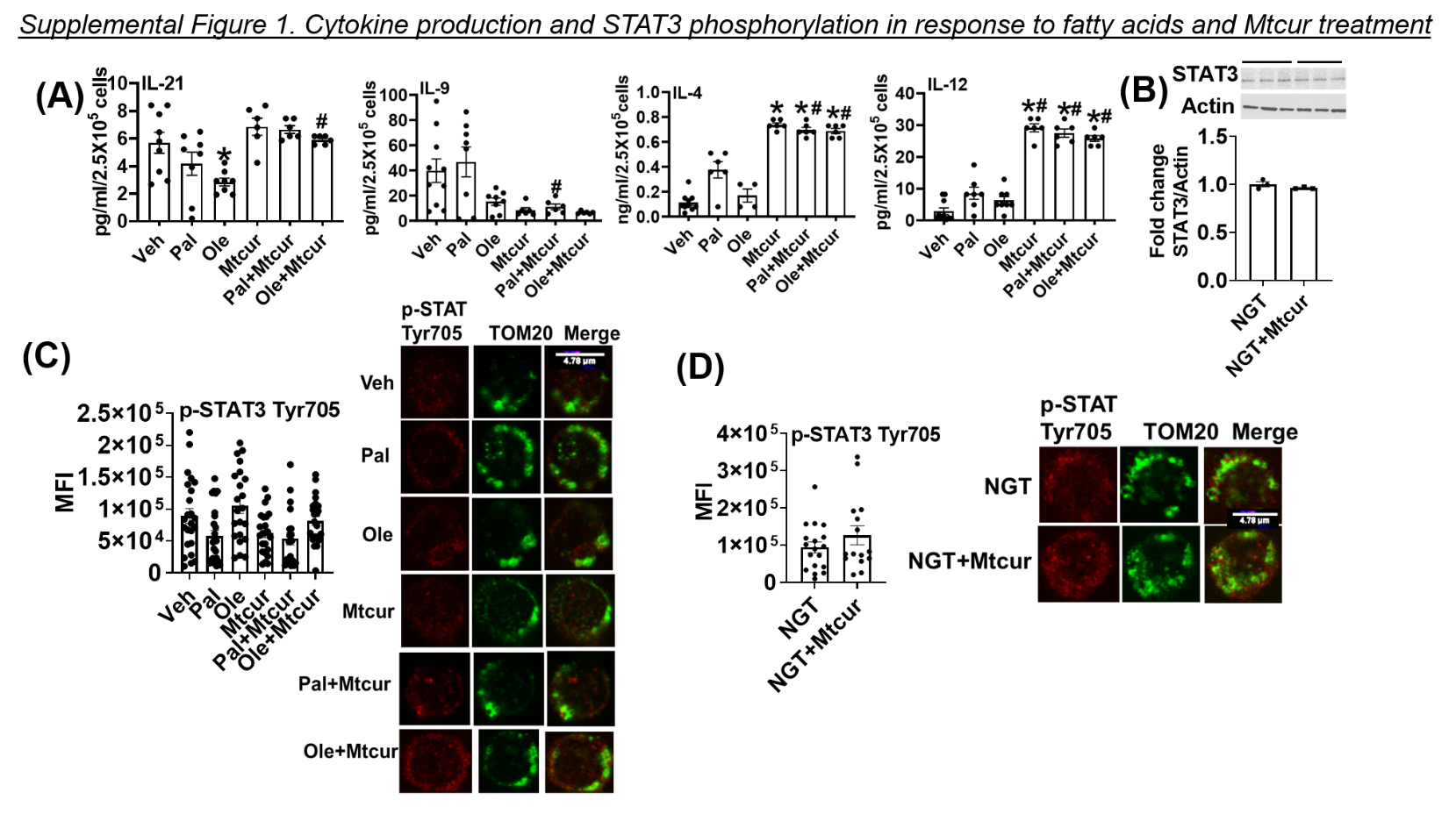
**Supplemental Materials:**

***Supplemental Figure 1: Pharmacological blockade of mitochondrial STAT3 alters cytokine profiles, without affecting STAT3 tyrosine phosphorylation***

**(A)**Cytokine production, **(B)**STAT3 expression **(C)**, p-Tyr 705 STAT3 expression in cells from lean and **(D)** NGT subjects after Mtcur treatment. N= 5-9 A, N=3 per group, B-D. Each N represents cells obtained from one subject. At least 7-10 fields per slide were imaged at 63Xmagnification with oil immersion, on a Zeiss LSM 800 confocal microscope. In fields where numerous cells/ field were observed, the averages are plotted. *p<0.05 vs. veh, # p<0.05 vs. respective veh, palmitate or oleate treatment.

Associated with main figure 2.


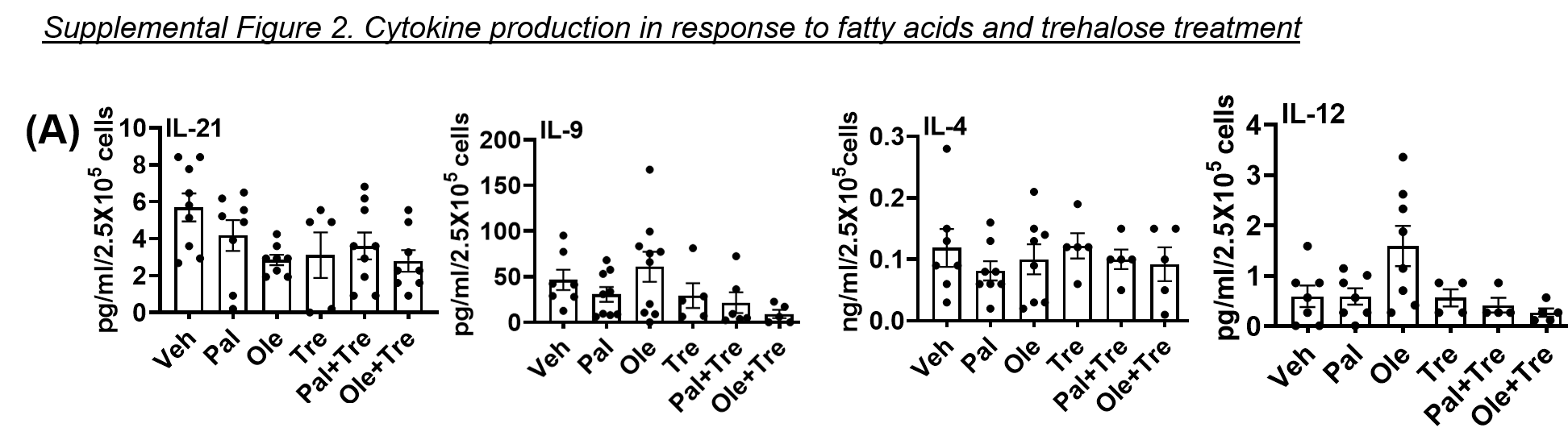


***Supplemental Figure 2: Cytokine production in cells after treatment with trehalose***

**(A)** Cytokine production after fatty acids and trehalose treatment measured via Luminex bioplex assay. N= 5-9, A. Each N represents cells obtained from one subject. Associated with main figures 3 and 4.


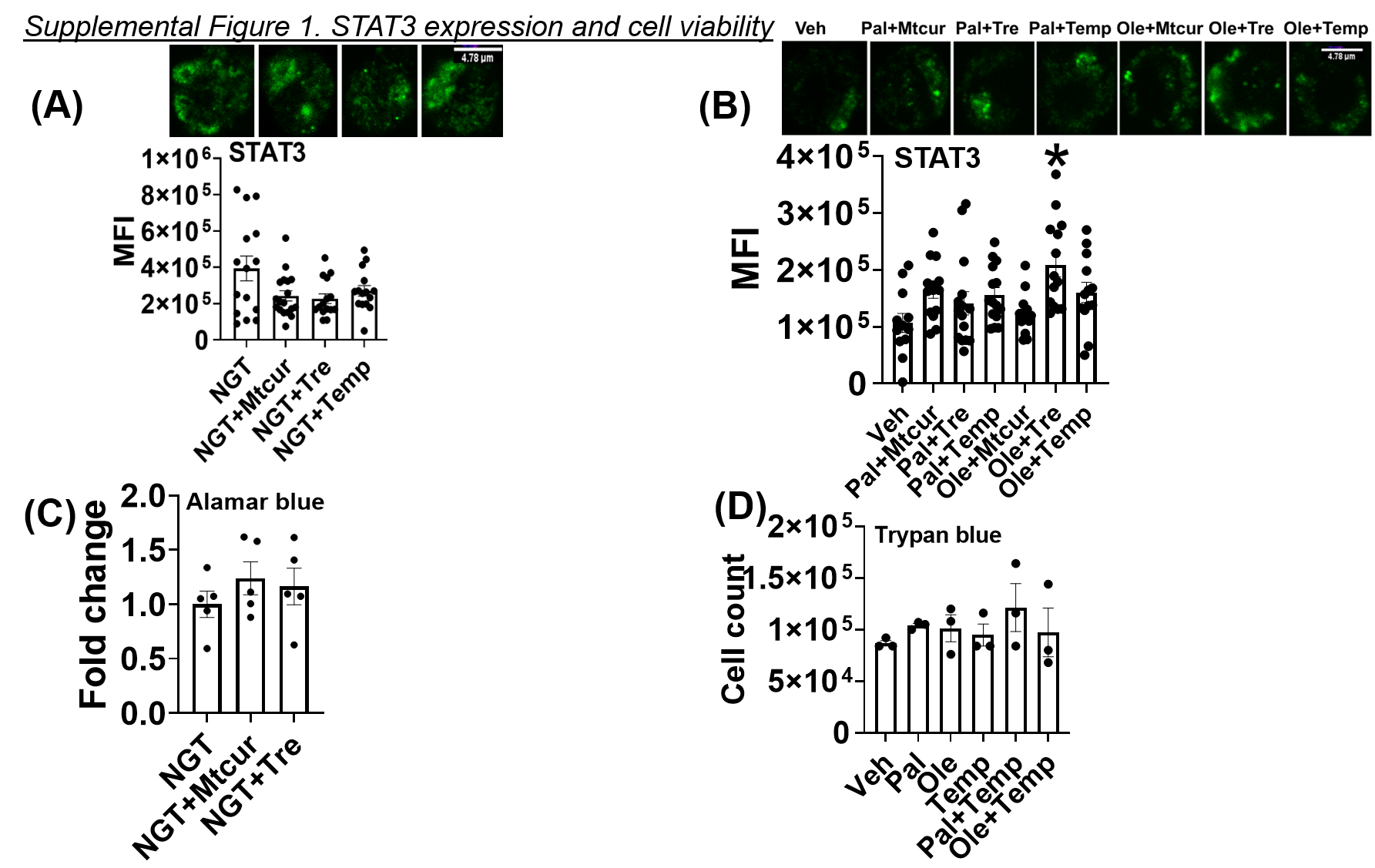


***Supplemental Figure 3: STAT3 expression and cell viability.***

1. STAT3 expression in NGT subjects, **(B)**STAT3 expression after fatty acid treatments **(C)** cell viability plotted as fold change in fluorescence after an alamar blue assay and **(D)** cell viability assessed via trypan blue assay. N=3 per group, A,B and D. N=5, C. Each N represents cells obtained from one subject. At least 7-10 fields per slide were imaged at 63Xmagnification with oil immersion, on a Zeiss LSM 800 confocal microscope. In fields where numerous cells/ field were observed, the averages are plotted. *p<0.05 vs. veh.

Associated with main figures 1-7
